# Pulmonary *Staphylococcus aureus* infection regulates breast cancer cell metastasis via neutrophil extracellular traps (NETs) formation

**DOI:** 10.1002/mco2.22

**Published:** 2020-07-30

**Authors:** Jia‐Long Qi, Jin‐Rong He, Cun‐Bao Liu, Shu‐Mei Jin, Rui‐Yu Gao, Xu Yang, Hong‐Mei Bai, Yan‐Bing Ma

**Affiliations:** ^1^ Chinese Academy of Medical Sciences and Peking Union Medical College Institute of Medical Biology Kunming China; ^2^ Department of Pharmacology Laboratory Yunnan Institute of Materia Medica NO24, LENGSHUITANG, BIJI ROAD, XISHAN QU Kunming 650000 China; ^3^ School of Basic Medical School Kunming Medical University Kunming China

**Keywords:** autophagy, cancer metastasis, multidrug‐resistant (MDR) bacterial, neutrophil extracellular traps (NETs), pneumonia

## Abstract

The formation of neutrophil extracellular traps (NETs) was recently identified as one of the most important processes for the maintenance of host tissue homeostasis in bacterial infection. Meanwhile, pneumonia infection has a poor effect on cancer patients receiving immunotherapy. Whether pneumonia‐mediated NETs increase lung metastasis remains unclear. In this study, we identified a critical role for multidrug‐resistant *Staphylococcus aureus* infection‐induced NETs in the regulation of cancer cell metastasis. Notably, *S. aureus* triggered autophagy‐dependent NETs formation in vitro and in vivo and increased cancer cell metastasis. Targeting autophagy effectively regulated NETs formation, which contributed to the control of cancer metastasis in vivo. Moreover, the degradation of NETs by DNase I significantly suppresses metastasis in lung. Our work offers novel insight into the mechanisms of metastasis induced by bacterial pneumonia and provides a potential therapeutic strategy for pneumonia‐related metastasis.

## Graphical abstract

Neutrophils help cancer cells metastasis in distant organs. Clinical multidrug‐resistant (MDR) *S. aureus* infection enhances cancer recurrence via orchestrating the NETosis network to promote metastasis. Targeting autophagy and/or NETs in bacterial infection may be useful for patient prognosis and may reduce metastasis.

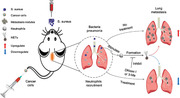

## 1 INTRODUCTION

Neutrophils are the first line of defense in the host immune system against pathogen infection and act as a double‐edged sword in the processes of cancer occurrence and development.^1^ A novel type of neutrophil‐programmed cell death that involves the formation of neutrophil extracellular traps (NETs) was recently identified as one of the most important processes in the maintenance of host tissue homeostasis.^2^ Although the capturing and killing of pathogens are the most important functions of NETs, they also participate in antiviral infection,^3^ the systemic inflammatory response,^4^ lung disease,^5^ autoimmune diseases,^6^ thrombosis,^7^ tumor metastasis,^8^, ^9^ and other functions.^10^ During NETs formation, neutrophils initiate cell death by slowly releasing cell contents, such as myeloperoxidase (MPO), neutrophil elastase (NE), DNA, and other molecules, which is the main pathway for cell death.^11^, ^12^ Furthermore, neutrophils also quickly secrete and release nuclear contents through exosomes, becoming NETs for the digestion of microorganisms.^13^ In recent years, more and more studies have shown that NETs are involved in cancer metastasis.^14^ The NE‐mediated degradation of E‐cadherin (E‐cad) promotes tumor progression and invasion under the stimulation of lipopolysaccharides (LPS), which is found in the outer membrane of all gram‐negative bacteria.^15^ MPO catalyzes chloride and H_2_O_2_, and the formation of the metabolite HOCl promotes epithelial cell damage and mutation by activating matrix metalloproteinase 2/9, which promote tumorigenesis and metastasis.^16^

Cancer metastasis is one of the leading causes of cancer‐related mortality worldwide, and approximately 13% of all tumor‐related deaths are related to metastasis. Currently, surgical treatment is one of the most effective strategies for cancer patients, and most cancer patients receive at least one surgical procedure as part of their treatment.^17^ Increased circulating tumor cells and intestinal obstruction or bacterial infections are the two most harmful side effects of surgical treatment. In some surgeries, >40% of patients suffer from pneumonia, peritonitis, sepsis, or severe postoperative infections, which are associated with poor tumor outcomes. Therefore, in addition to infectious diseases, postoperative infections can also lead to tumor recurrence and metastasis.^18^ NETs can be detected after tumor resection, and the abundance of NETs enhances the risk of tumor recurrence.^18^ The inflammatory state of individual peripheral organs of cancer induced by NETs may be an important factor leading to tumor metastasis.^19^ Additionally, ovarian tumors recruit neutrophils, which are necessary for the colonization and entrapment of circulating tumor cells in the omental niche, and promote NETs formation.^20^

Multidrug‐resistant (MDR) *Staphylococcus aureus* is a gram‐positive, common pathogen for nosocomial bacteria that induces pneumonia, sepsis, and bacteremia, especially among intensive care unit patients.^21^ The growth of MDR *S. aureus* infection has been recognized as one of the most urgent public health threats not only because it is resistant to all commonly used antibiotics^22^ but also because its α toxin (AT) improves the dissemination and propagation of most gram‐negative bacteria.^23^ In addition, *S. aureus* infection mediates the enhancement of nonsmall cell lung cancer cell metastasis due to upregulation of the TLR4/MyD88 pathway.^24^ Recent studies have conclusively demonstrated that *S. aureus* biofilms release leukocidins^25^ and that AT can induce the rapid formation of NETs.^26^, ^27^ Previous work by us and others has demonstrated that pulmonary gram‐negative bacterial infection or cigarette stimulation can induce NETs in vitro and in vivo.^28^, ^29^ However, the role of bacterial infection‐induced NETs in cancer cell metastasis remains unclear.

Herein, we identified a novel role for pulmonary bacterial infection in NETs formation in cancer cell metastasis that is regulated by the progression of autophagy. *Staphylococcus aureus* infection recruited neutrophils into the lung tissues and triggered cell death in a NETs‐like forms. Exogenous treatment with the autophagy inhibitor 3‐methyladenine (3‐MA) or DNase I significantly reduced cancer cell metastasis and response to MDR *S. aureus* infection. Thus, our study describes a novel mechanism by which bacteria‐mediated NETs promote cancer cell metastasis, providing insight into the role of autophagy in regulating NETs formation during gram‐positive *S. aureus* infection, and provides potential targets for regulating cancer cell metastasis during MDR bacterial infection.

## 2 RESULTS

### 2.1 Breast cancer metastasis induced neutrophil accumulation in the lung

**FIGURE 1:**
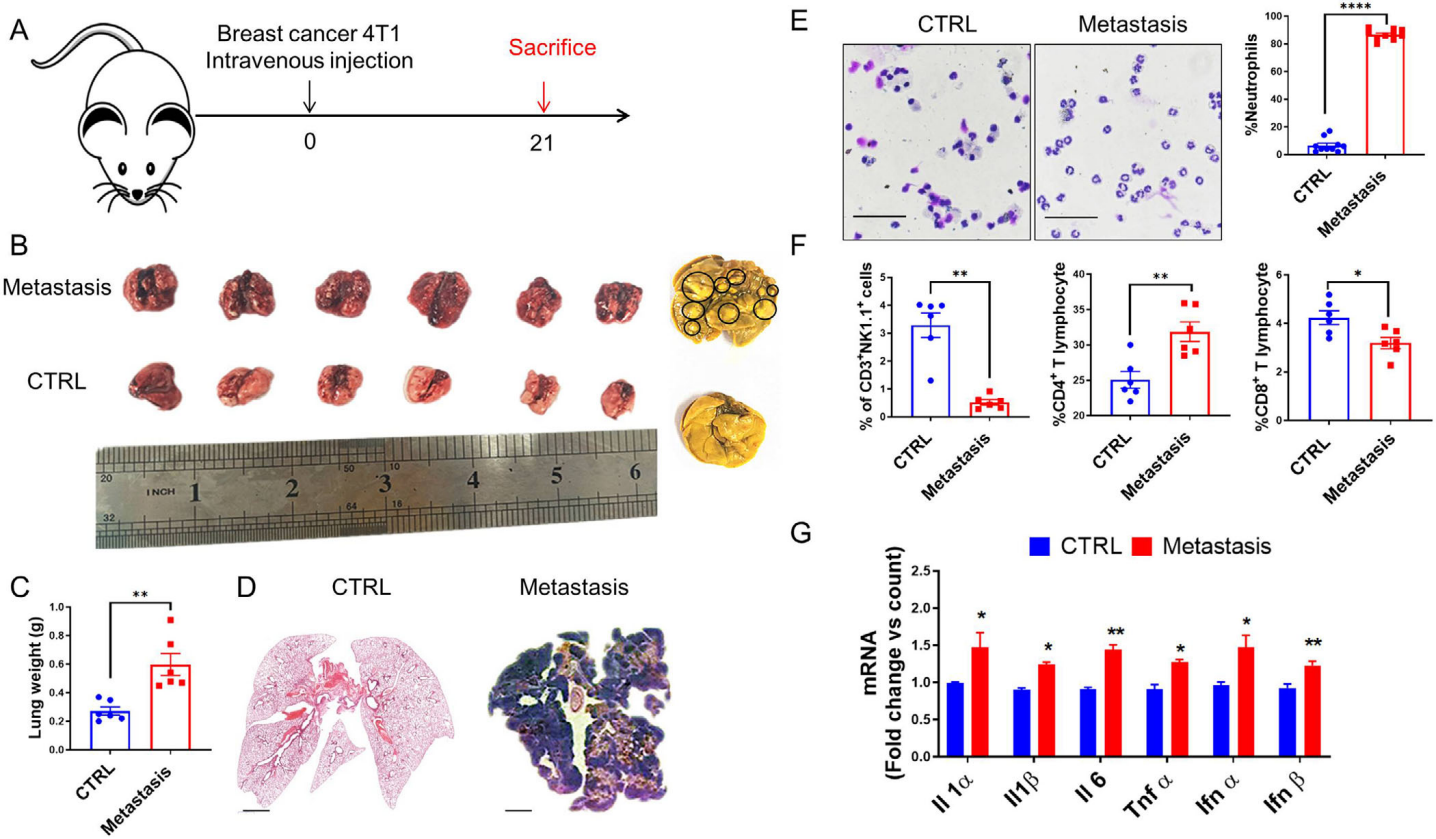
The lung immune cells profile was changed in a breast cancer lung metastasis model. A, A schematic diagram of murine breast cancer metastasis model construction. Mice (n = 6) were intravenously injected with the murine breast cancer cell line 4T1 (1 × 10^5^ cells per mouse). At day 21 after incubation, all mice were sacrificed. B, Representative images of lung tissues from both groups. The black circle represented the metastasis nodules on the lung surface. C, The weight of lung tissue in control and metastatic mice is presented. D, Representative images of whole lung tissue by H&E staining. E, Lung neutrophil identification and Swiss‐Giemsa staining in bronchoalveolar lavage fluid (BALF) cells. F, Flow cytometry analysis of immune cells in the BALF. BALF cells were stained with CD3‐APC, NK1.1‐PE, APC‐CD8α, and PE‐CD4 for the identification of immune cell profiles. G, Gene expression of inflammatory cytokines in the lung tissues of CTRL and metastatic mice. Data represent three independent experiments and are shown as the mean ± standard error of the mean (SEM). ^*^ *P *< .05, ^**^ *P *< .01, and ^****^ *P *< .0001. Scale bars, 50 and 1000 µm

Nearly 90% of breast cancer‐related deaths are due to metastasis, especially in the lungs and bones.^30^ To generate a breast cancer metastasis model, we intravenously injected breast cancer 4T1 cells at a dose of 1 × 10^5^ cells into each mouse, and mice were monitored until death. Lung metastasis occurred approximately 2 weeks after 4T1 cell injection, and all mice were sacrificed at day 21 for further analysis (Figure 1A). Obvious metastatic nodules were observed on the surface of lung tissues (Figure 1B), and the weights of the lung significantly increased (Figure 1C). Whole lung tissue hematoxylin and eosin (H&E) staining showed that the entire lung was filled with multiple metastatic nodules compared with the control lung tissues (Figure 1D). To better explore the role of lung microenvironment for metastasis, bronchoalveolar lavage fluid (BALF) cells were harvested and analyzed by cytospins at the endpoint. Obviously, neutrophils were recruited and enriched compared with control groups (Figure 1E). Furthermore, upon examining the composition of the immune cells, including natural killer (NK) cells, lymphocytes, eosinophils, basophils, CD4^+^ T cells, and CD8^+^ cells, in the BALF, antitumor NK cells and CD8^+^ T cells were reduced, and tumor‐promoting neutrophils and CD4^+^ T cells were increased (Figure 1F). In addition to immune cells infiltration, inflammatory cytokine production also induces an immunosuppressive microenvironment that creates a hotbed for tumor metastasis.^19^ Thus, we analyzed genes encoding pro‐inflammatory cytokines in the lung. After metastasis, pro‐inflammatory cytokines, including Il1α, Il1β, Il6, Il18, and TNF‐α, were significantly induced in lung metastases, indicating that inflammatory cytokines may play a positive role in cancer metastasis. Moreover, type I interferon gene (IFN‐α and IFN‐β) expression was also induced (Figure 1G). Taken together, these results indicate that neutrophils infiltration and inflammatory cytokines production may positively affect cancer metastasis.

### 2.2 Neutrophils play positive roles in breast cancer metastasis

**FIGURE 2:**
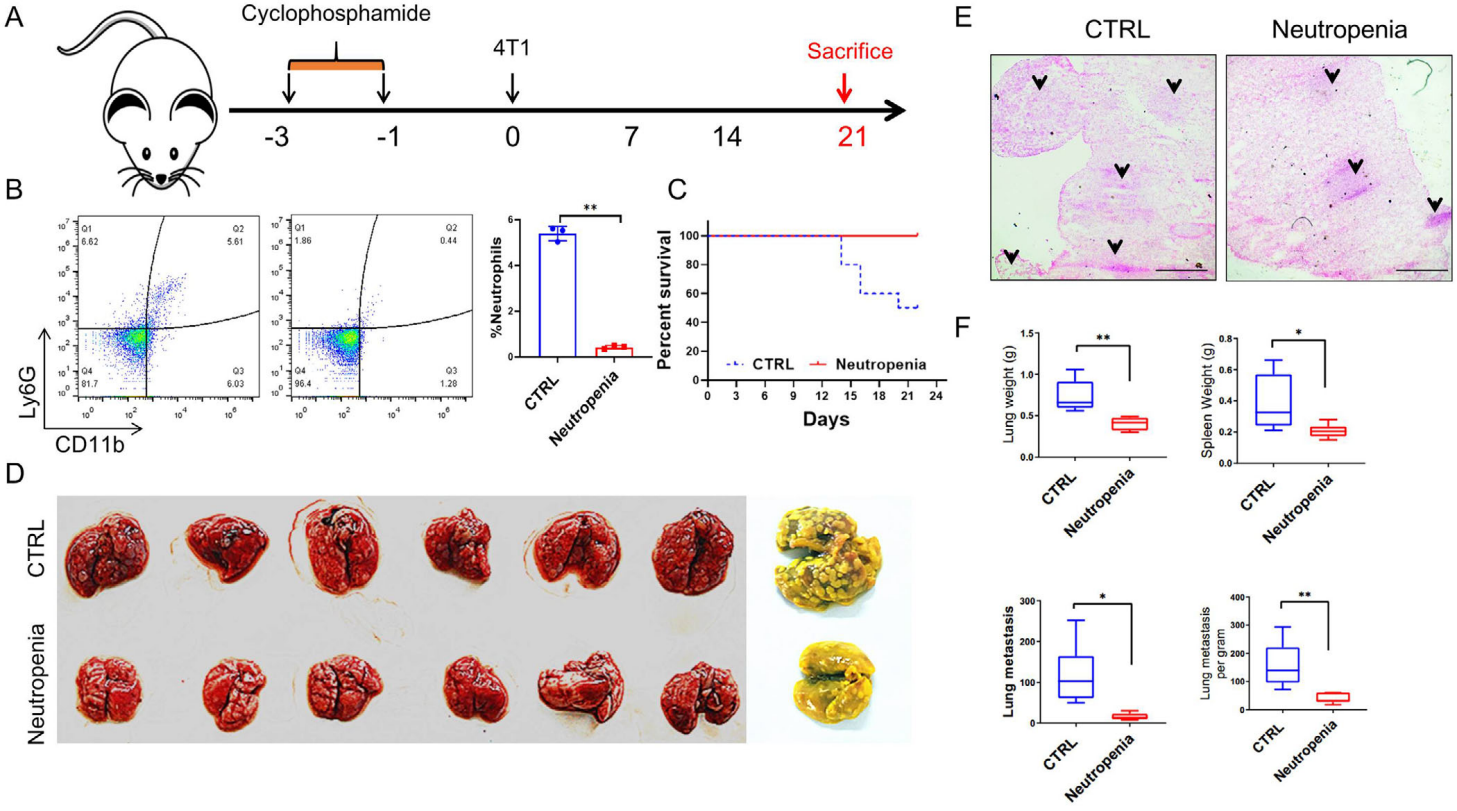
Neutrophil depletion decreases breast cancer metastasis. A, A schematic diagram of mice neutrophil depletion in cancer metastasis. Mice were intraperitoneally injected with cyclophosphamide for neutrophil depletion, and then 4T1 cells were intravenously injected. B, Flow cytometry analysis of neutrophils in the BALF with antimouse CD11b‐APC and Ly6G‐PE. C, The survival rate of mice in both CTRL and neutropenic group. Mice in CTRL (n = 6) and neutropenic mice (n = 6) were intravenously injected with 4T1 cells, and the survival rate was monitored. D, Representative images of lung tissues from both groups. All lung tissues were harvested and fixed with Bouin's buffer. E, Representative images of left lung tissue with H&E staining. The black arrows indicate metastasis nodules. F, Histogram analysis of the lung weights, spleen weights, and lung metastatic nodules was monitored. Scale bars, 100 µm. The data represent three independent experiments and are shown as the mean ± SEM. ^*^ *P *< .05, ^**^ *P *< .01, ^***^ *P *< .001, and ^****^ *P *< .0001

Neutrophils act as a double‐edged sword in the processes of cancer occurrence and development, which depend on the neutrophil phenotype.^31^ To investigate whether neutrophils facilitate tumor cell implantation into the lung, we used cyclophosphamide to eliminate neutrophils in the model. Mice were intranasally administered 1 mg/kg cyclophosphamide on day 3 and day 1 before 4T1 cell injection (Figure 2A). The number of BALF CD11b^+^Gr‐1^+^ neutrophils was significantly reduced after 2 weeks (Figure 2B). These results suggest that we successfully established a neutropenia model. To determine the effect of the absence of neutrophils on tumor metastasis, we further injected 4T1 cells into neutropenic mice. As expected, more wild‐type (WT) mice suffered from severe symptoms of illness, such as a hunched back and ruffled fur, than did neutropenic mice. As a result, all neutropenic mice all survived, whereas only 50% of WT mice survived beyond day 22 (Figure 2C). To confirm whether the reduced mortality observed in WT mice was due to cancer cell lung metastasis, mice were sacrificed, and lung tissues were fixed and stained with Bouin's fixative solution. In addition, we enumerated the metastatic nodules in the lung, and the lung metastasis burden in WT mice was more serious than that in neutropenic mice (Figure 2D). H&E staining showed that lung metastatic nodules per vision were also significantly increased in quantity compared with that in neutropenic mice (Figure 2E). Consistent with these results, the lung and spleen weights were also reduced (Figure 2F). To accurately explore the role of neutrophils in metastasis, we further use anti‐Ly6G to deplete neutrophils in vivo. No surprise, lung metastasis was suppressed in neutrophils depleting group (Figure S1A‐C). Collectively, these results indicate that neutrophils promote tumor metastasis.

### 2.3 Pulmonary *S. aureus* infection increases tumor metastasis

**FIGURE 3:**
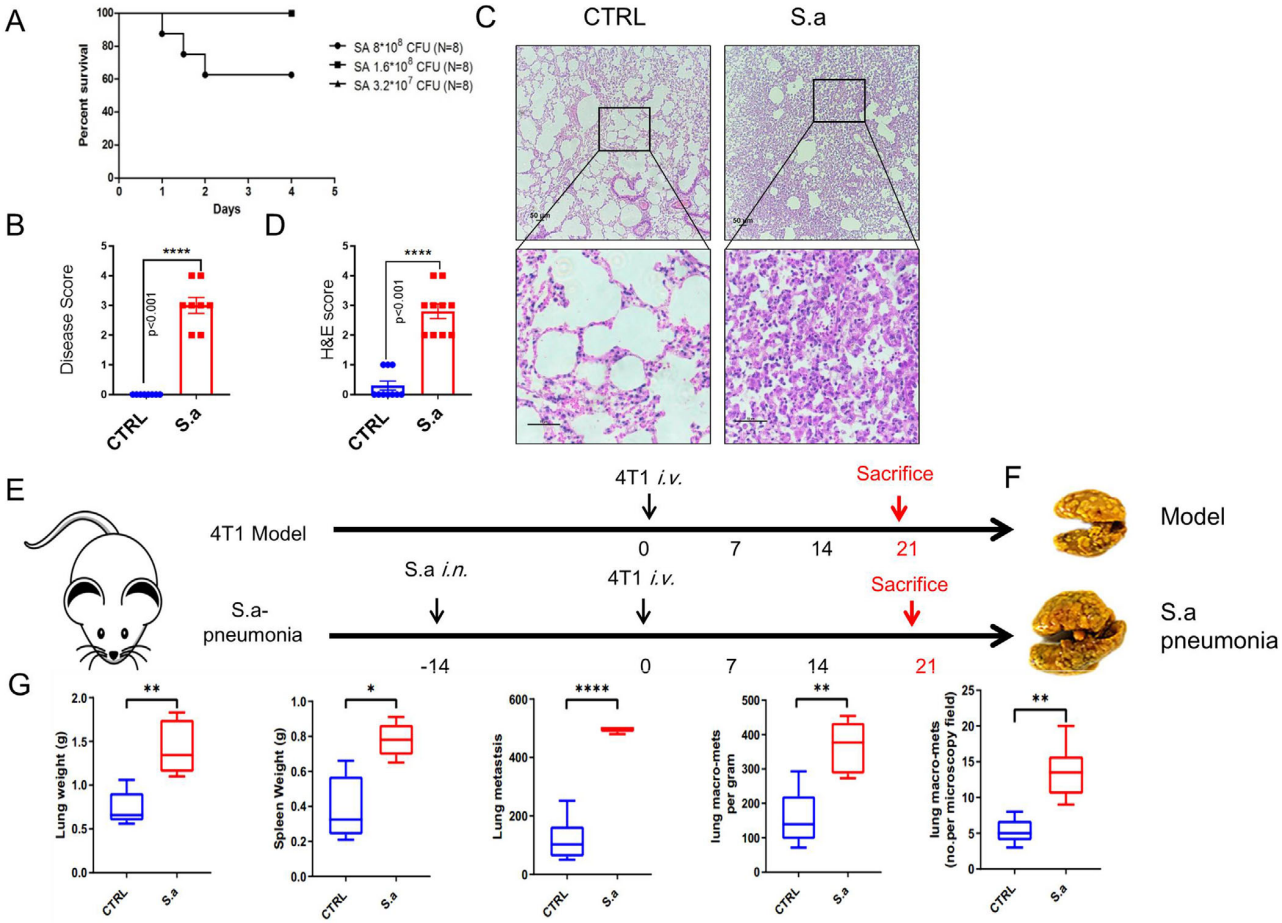
*Staphylococcus aureus* infection increased pulmonary metastasis. A, The survival rate of mice infected with *S. aureus*. Mice (n = 8) were intranasally infected with increasing doses of *S. aureus*, and the survival rate was monitored. B, Disease scores were recorded 12 h after a sublethal dose of *S. aureus* infection. C, Representative images of left lung tissues from CTRL and *S. aureus* infection groups by H&E staining. D, Results of H&E score. E, A schematic diagram of bacterial pneumonia promotes metastasis. Two weeks after mice intranasally infected with a sublethal dose of *S. aureus*, 4T1 cells were intravenously administrated and 21 days after implanting, all mice were sacrificed. F, Whole lung tissues and representative fixed lung were shown. Lung tissues of pneumonic and CTRL mice were harvested and fixed with Bouin's buffer. G, The weights of the lung, spleen, and lung metastatic nodules were monitored. Statistical analysis was performed by Student's paired *t*‐test. Scale bars, 50 µm. The data represent three independent experiments and are shown as the mean ± SEM. ^*^ *P *< .05, ^**^ *P *< .01, and ^****^ *P *< .0001

*Staphylococcus aureus* infection has been recognized as one of the most urgent public health threats because of its resistance to all commonly used antibiotics.^23^ More seriously, pneumonia infection has been reported to have a poor effect on cancer patients receiving immunotherapy.^32^ To examine whether *S. aureus* infection increases tumor metastasis, we established a model of pneumonia infection by intranasally administering bacteria. In mice infected with bacteria, the mortality rate increased in a dose‐dependent manner (Figure 3A), and we chose the lowest sublethal dose of 3.2 × 10^7^ colony‐forming units (CFUs) per mouse for further analysis. To confirm the symptoms of pneumonia induced by bacterial infection, we recorded the disease score in uninfected and bacteria‐infected mice 10 h after treatment.^33^ Bacteria‐infected mice suffered from pathological symptoms such as ruffled fur, a hunched back, and rapid breathing (Figure 3B). Consistent with these results, we performed H&E staining to examine lung tissue immune cells infiltration, which was significantly higher in bacteria‐treated mice than in untreated mice including neutrophils (Figures 3C, 3D, and S4). Taken together, these findings indicate that the intranasal administration of bacteria establishes murine pneumonia. To determine whether murine pneumonia increased metastasis, mice were intravenously injected with 4T1 cells 2 week after pneumonia establishment (Figure 1E). Lung tissues were harvested at the endpoints; we found that lung metastasis was increased in the pneumonia group compared to model group (Figure 3F). The weights of the lung, spleen, and metastatic nodules were significantly increased (Figure 3G). Thus, these results indicate that *S. aureus* infection induces immune cells infiltration and increases cancer cell metastasis.

### 2.4 *Staphylococcus aureus* regulates NETosis in vitro and in vivo

**FIGURE 4:**
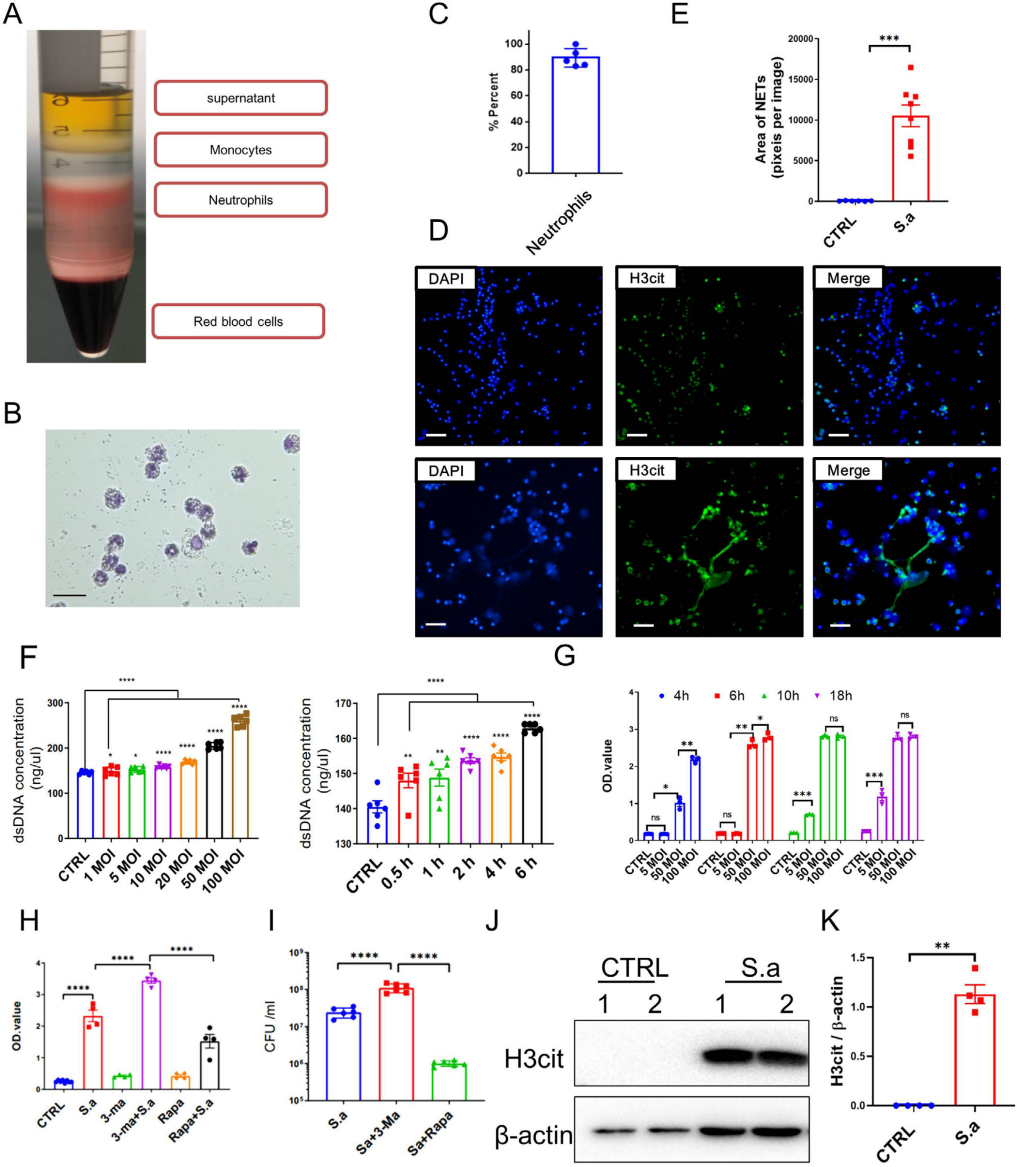
*Staphylococcus aureus* infection promotes NETs formation in vitro and in vivo. A, Neutrophils were separated from mouse peripheral blood mononuclear cells (PBMCs) by density gradient centrifugation. B, Neutrophils were identified and stained with Swiss‐Giemsa buffer. C, Histogram analysis of neutrophils purity. The purity of neutrophils was determined in five different sections, each of which was recorded from 10 independent sections. D, Representative images of *S. aureus*‐triggered NETs formation in vitro. Purified neutrophils were infected with or without *S. aureus* for 6 h, and then stained with DAPI and anti‐H3cit for NETs formation identification in vitro. E, The quantification results of NETs areas by ImageJ. F, XTT cell viability assay to determine the cell death of neutrophils treated with *S. aureus*. Neutrophils cytotoxicity was analyzed following *S. aureus* infection in a time‐ and dose‐dependent manner. G, Neutrophil‐free dsDNA was increased under *S. aureus* infection. Neutrophil was stimulated with *S. aureus* in dose‐ and time‐dependent manner, supernatants were harvested, and free dsDNA was detected by PicoGreen dsDNA detection kit. H, Lactate dehydrogenase (LDH) assay of neutrophil pretreated with the autophagy inhibitor 3‐MA and the agonist rapamycin. I, Bacterial burden in neutrophil supernatants was detected following 3‐MA and rapamycin treatment. J, Immunoblotting analysis of *S. aureus*‐triggered NETs formation in vivo. Mice were treated with a sublethal dose of *S. aureus*, and lung tissues were harvested and analyzed with H3cit. K, The quantification analysis of H3cit band. Statistical analysis was performed by Student's paired *t*‐test between two groups, and one‐way ANOVA for three or more groups. Scale bars, 50 µm. The data represent three independent experiments and are shown as the mean ± SEM. ^*^ *P *< .05, ^**^ *P *< .01, ^***^ *P *< .001, and ^****^ *P *< .0001

Neutrophils are the first line of defense in the host immune system against pathogen infection, and in recent years, NETs have been identified to play an important role in bacterial clearance.^2^ To examine *S. aureus* infection‐induced cell death in neutrophils, we separated neutrophils from peripheral blood mononuclear cells (PBMCs) derived from healthy mice (Figure 4A). The purity of the neutrophils was determined with cytospins, and cells were stained with Wright‐Giemsa (Figures 4B and 4C). The neutrophils with high purity and activity were obtained for further analyses. To determine whether *S. aureus* infection increased NETs formation, neutrophils were infected with a multiplicity of infection (MOI) of 50 bacteria for 6 h. Strikingly, *S. aureus* enhanced H3cit expression, which is a hallmark for NETs, under *S. aureus* treatment (Figures 4D and 4E). Consistent with these results, we found that *S. aureus* infection induced neutrophils releasing free dsDNA in a dose‐ and time‐dependent manner by a PicoGreen assay (Figure 4F). In addition, neutrophil cell death was also examined with the XTT cell viability kit. Neutrophil death was increased in a time‐ and dose‐dependent manner (Figure 4G). Taken together, *S. aureus* infection triggered NETs‐like death in vitro. Previous studies have reported that the occurrence of NETs by gram‐negative bacteria was dependent on the process of autophagy.^28^ To investigate the role of autophagy in response to gram‐positive *S. aureus*‐induced NETs, neutrophils were pretreated with the autophagy inhibitor 3‐MA and the agonist rapamycin (Rapa) before bacterial infection. Cell death was significantly increased and bacterial clearance ability was significantly reduced in 3‐MA‐treated neutrophils, whereas the effects were reversed in neutrophils under rapamycin treatment (Figures 4H and 4I). Furthermore, we also analyzed the formation of NETs in vivo. Immunoblotting revealed that the expression of H3Cit, which represents NETs in response to *S. aureus* infection, was lower in untreated mice (Figure 4J and 4K). Interestingly, we also observed co‐localization signals in the metastatic nodules (Figure S2). To confirm this phenomenon, we analyzed NETs formation in another mouse tumor model. Mice were implanted with TC‐1 cells, and tumor tissues were then collected at the indicated times. Intratumoral NETs were identified in a tumor development process‐dependent manner (Figure S3), which demonstrated that NETs formation not only promotes metastasis but also increased tumor growth. Taken together, these results suggest that gram‐positive *S. aureus* infection increases NETs formation in vitro and in vivo and the formation is dependent on the process of autophagy.

### 2.5 Autophagy controls pneumonia‐induced NETs‐mediated metastasis

**FIGURE 5:**
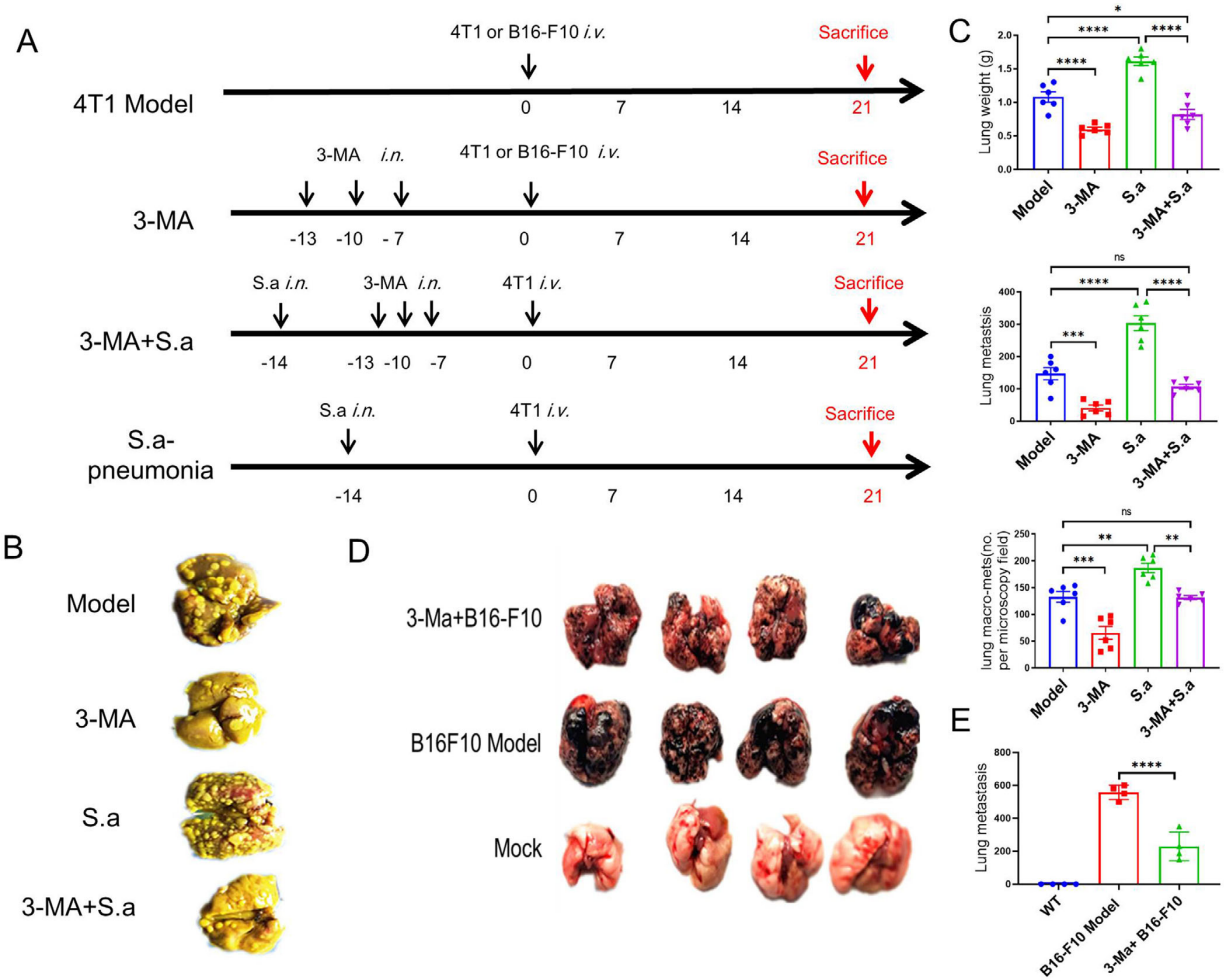
Pretreatment with 3‐MA inhibits cancer metastasis. A, A schematic diagram of 3‐MA‐inhibited cancer metastasis. After bacterial infection, 3‐MA was continuously administrated for three times, and then lung metastasis was determined among four groups, Model, 3‐MA, 3‐MA plus Sa, and Sa pneumonia group. B, Representative images of lung tissues. Lung tissues were harvested and fixed with Bouin's buffer. C, The weights of the lung, spleen, and lung metastatic nodules were monitored. D, 3‐MA inhibits B16 ‐F10 lung metastasis in vivo. E, The quantification results of B16‐F10 metastasis nodules on the lung surface. Statistical analysis was performed by Student's paired *t*‐test between two groups. Data represent three independent experiments and are shown as the mean ± SEM. ^*^ *P *< .05, ^**^ *P *< .01, ^***^ *P *< .001, and ^****^ *P *< .0001

Although autophagy can effectively control bacterial infection‐induced NETs formation in vitro, whether this effect regulates and/or controls the increase in metastasis caused by bacterial infection in vivo remains unknown. To address these questions, we treated mice with 3‐MA after bacterial infection and then 4T1 cells were administrated (Figure 5A). At the endpoint of the experiments, lung tissues were harvested and stained with Bouin's buffer (Figure 5B). Strikingly, the weights of lung tissues were lower in 3‐MA‐treated groups than that in nontreated groups, and lung metastatic nodules were also decreased in 3‐MA‐treated groups (Figure 5C). To confirm the effects of autophagy inhibitor in metastasis, a murine melanoma cell line, B16‐F10, was also employed. In line with these results, we found that 3‐MA also suppresses B16‐F10 in vivo and lung metastatic nodules were sharply decreased (Figures 5D and 5E). Thus, these results indicated that autophagy was participated in NETs‐mediated metastasis in vivo.

### 2.6 Treatment with DNase I attenuates NETs‐mediated metastasis

**FIGURE 6:**
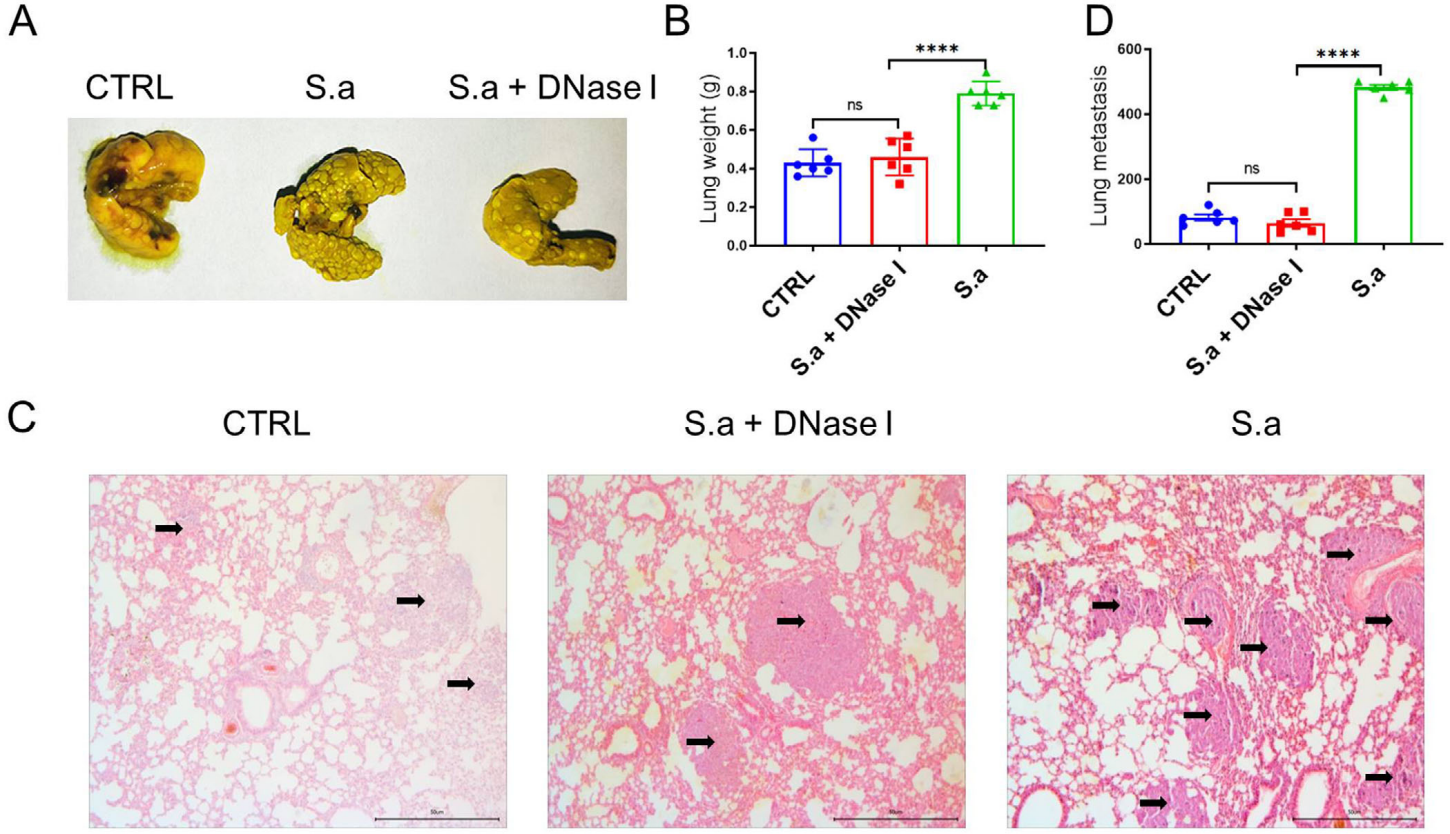
DNase I suppressed metastasis under *S. aureus* infection in vivo. A, Representative images of lung tissues with 4T1 metastasis. Mice were treated with or without DNase I after *S. aureus* stimulation, and then 4T1 cells were injected. B, The results of lung weight and metastasis nudes in each groups were monitored. C, Representative H&E staining images of lung left lobe. Blank arrows represented metastasis nudes. Statistical analysis was performed by Student's paired *t*‐test between two groups, and one‐way ANOVA for three or more groups. Scale bars, 50 µm. Data represent three independent experiments and shown as mean ± SEM. ^****^ *P *< .0001

It is well known that autophagy is closely related to NETs formation, and in this paper, we found that treatment with 3‐MA significantly suppressed NETs‐mediated metastasis. To more directly demonstrate that bacterial pneumonia‐induced NETs promote cancer metastasis, we administrated mice with DNase I, a specific degradation for NETs‐DNA structure, after *S. aureus* infection. No surprise, lung metastasis in the pneumonia group was increased and that this phenomenon was reversed in DNase I‐treated group (Figure 6A). No significant difference between the model group and DNase I‐treated group was found, indicating that DNase I could only influence pneumonia NETs‐mediated metastasis. In addition, lung weights were lighter in DNase I‐treated group than in pneumonia groups (Figure 6B), along with fewer metastasis nodules per section by H&E staining. Taken together, the treatment of DNase I significantly suppresses bacteria‐triggered NETs.

## 3 DISCUSSION

**FIGURE 7:**
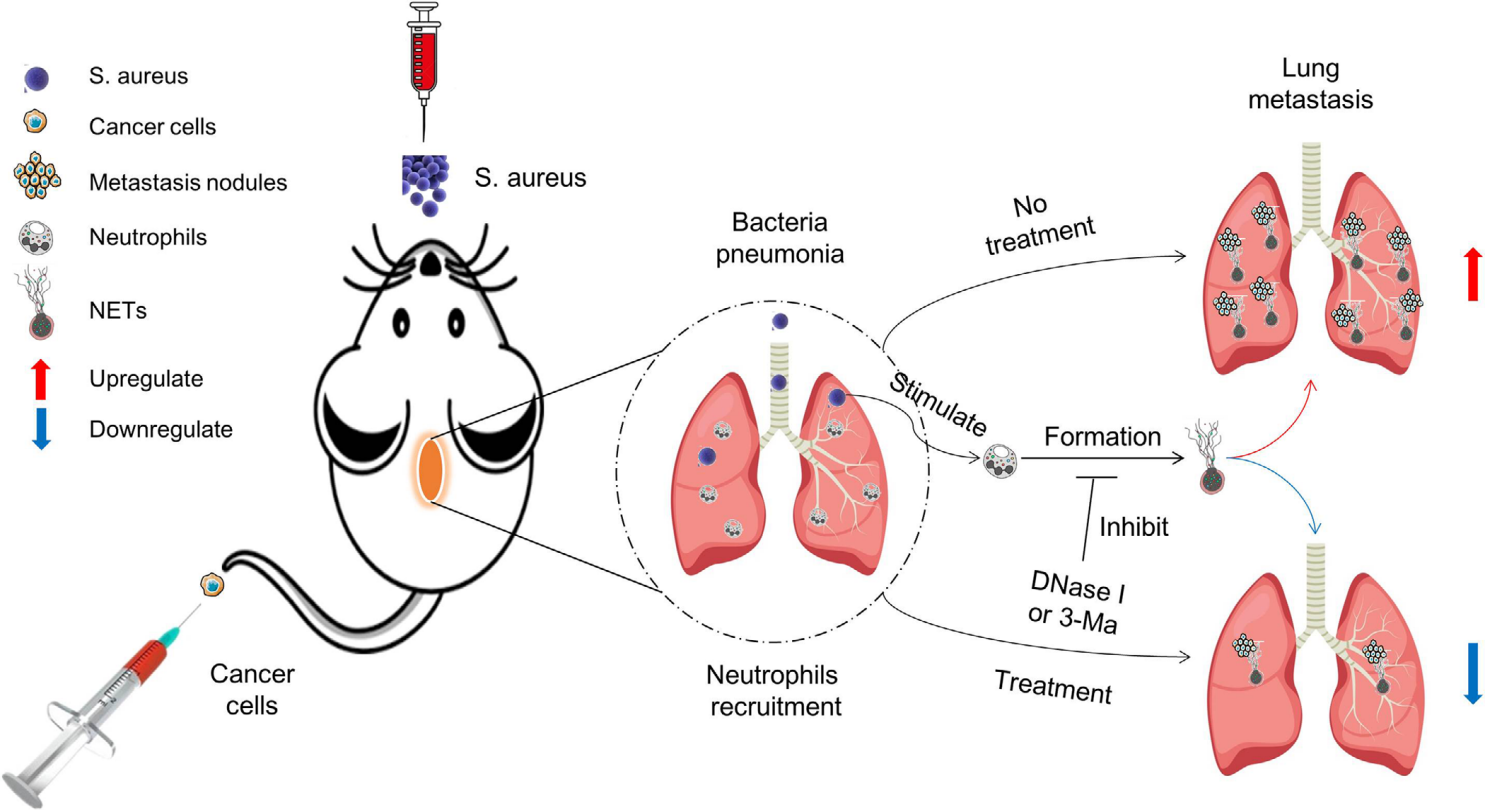
Schematic diagram of bacterial pneumonia‐mediated NETs that promote cancer metastasis. Neutrophils help cancer metastasis in many ways. During *S. aureus* infection, neutrophils were recruited into lung tissues and triggered NETs formation, which trapped circulated cancer cells and promoted new metastasis. Autophagy was taken part in NETs formation and bacterial clearance. Meanwhile, targeting of autophagy or treatment of DNase I, resulting in NETs degradation, reduced cancer metastasis

There are increasing studies that show that bacterial and cancer cells have a co‐existing relationship in patients and even helpful for metastasis, simultaneously. This has important clinical guidance significance for cancer patients preventing metastasis. However, the molecular mechanism of how bacterial infection results in distant metastasis is still not well understand. In this study, we have demonstrated the negative role of MDR bacterial infection‐triggered pneumonia in cancer metastasis due to NETs formation. The formation of NETs has been identified as one of the most important strategies for host neutrophils against pathogenic bacterial infection.^34^ During bacterial infection, NETs capture and kill bacteria to eliminate pathogen amplification and damage to the host.^28^ And the residual NETs also cause a sustained inflammatory response that is harmful for normal tissue, especially in cancer patients.^14^ In this study, we found that breast cancer lung metastasis influences immune cell profiles, particularly neutrophils in lung BALF, which is essential for cancer metastasis. *Staphylococcus aureus* stimulation recruits neutrophils into lung tissues and triggers neutrophils cell death into a NETs structure in vitro and in vivo. Furthermore, we demonstrate that NETs structure is crucial for cancer metastasis. Here, we show that pulmonary bacterial infection‐induced NETs formation is a negative regulator in cancer recurrence and metastasis (Figure 7), which are regulated by autophagy.

Currently, surgery, radiotherapy, chemotherapy, and immune checkpoint therapy are indispensable for cancer treatment. However, it has been reported that cancer metastasis and recurrence are the most urgent problems and account for 90% of all cancer‐related deaths.^30^ Until now, more and more demonstrated theories have been identified related to cancer metastasis: (a) cancer cells can escape from host immune surveillance via exosome and/or secreted proteins; (b) downregulated T‐cell response triggered immune weakening; (c) defects in antigen processing; (d) the function of immune‐suppressing cells and molecules in the tumor microenvironment; (e) postoperative pressure; and (f) gut and lung microbiome. For example, cancer cell chromosomal instability promotes cytosolic DNA leakage by activating the cGAS‐STING pathway response and promoting an inflammatory microenvironment that increases the occurrence of cancer metastasis.^35^ Circulating tumor cell‐neutrophils clusters gain the ability to form subpopulations by regulating cell cycle progression.^36^ During cancer processing, neutrophils are sounded with soiled tumor tissues, along with macrophages and other immune cells, consisting the immune cell profiles in tumor microenvironment, which is related to the prognosis of cancer patients. Neutrophils located in lungs trigger leukotrienes production, which results in co‐localization with selectively propagating cancer cells and promote metastasis.^37^ Neutrophils also dampen antitumor T cell immunity by suppressing the function of cytotoxic T lymphocytes.^38^ In our study, we established the murine breast cancer metastatic model and found that the number of neutrophils was enriched in the lungs, readjusting the immune cell profiles, and that lung inflammatory cytokines were significantly increased during metastasis (Figure 1). Additionally, we also found that neutrophils depleting by cyclophosphamide (Figure 2) or anti‐Ly6G (Figure S1) sharply reduced cancer metastasis, indicating the indispensable role of neutrophils in cancer metastasis.

NETs treatment, a type of neutrophils programmed cell death, is an important process to maintain host tissue homeostasis. During NETs treatment, neutrophils could quickly secrete and release nuclear contents through exosomes, forming a network‐like structure. A previous study reported that cigarette smoke extract‐ or LPS‐induced lung NETs produced during inflammation awakened dormant cancer cells in mice.^39^ However, there are now increasing studies that have reported that clearance of dormant cancer cells can be effective therapeutic strategies for inhibiting cancer recurrence. An inhibitor of PERK, which is highly expressed in dormant cancer cells, can kill cancer cells in vivo.^40^ In our study, we found that a sublethal dose of *S. aureus* infection increases cancer cell metastasis and the lung weights (Figure 3E‐G). In line with these results, we found that bacterial infection induced pneumonia in mice, resulting in ill symptoms, and increased immune cells infiltration and inflammatory cytokine production (Figures 3A‐D and S4), suggesting that lung metastasis enhancement is triggered by *S. aureus* infection. Furthermore, gram‐negative bacterial infection recruits and stimulates neutrophils into NETosis in vitro.^28^ Here, we showed that *S. aureus* infection triggered neutrophil cell death in a time‐ and dose‐dependent manner following the detection of NETs (Figure 4F‐I). Consistent with these results, we also found that NETs were occurred in lung after *S. aureus* infection (Figure 4J). Thus, our study reveals that *S. aureus* infection causes the recruitment of neutrophils into the lung and prompts NETs‐like cell death pathways that provide a hotbed for tumor metastasis.

Our recent finding and others identified that autophagy process is important for gram‐negative bacteria‐triggered NETs formation,^28^ which might affect cancer metastasis. However, the mechanism by which autophagy attenuates bacterial infection‐induced cancer cell metastasis remains obscure. Our study demonstrates that the autophagy inhibitor 3‐MA specifically inhibits cancer cell metastasis in vivo (Figure 5). After the specificity treatment with 3‐MA, lung metastatic nodules are decreased along with the lung weights in both breast cancer and melanoma model. Our results reveal a previously unrecognized functional role for autophagy in negatively regulating cancer cell metastasis, which is increased by gram‐positive bacterial *S. aureus* infection. We provide a rationale for exploring autophagy as a potential drug target for controlling cancer cell metastasis. It is well known that target autophagy is just an indirect way of regulating NETs formation. DNase has been widely employed in controlling inflammatory diseases via NETs degradation directly.^41^ In our study, blocking NETs by DNase treatment significantly suppressed bacterial pneumonia‐mediated metastasis enhancement but has no effects on other subjects, which demonstrated that the NETs formation by bacterial infection was the key event related to cancer metastasis enhancement (Figure 6). Taken together, our data support the hypothesis that bacterial pneumonia‐mediated NETs formation promotes metastasis.

## 4 CONCLUSION

In summary, we demonstrated that pulmonary bacterial infection enhances breast cancer metastasis due to the formation of NETs that trap circulating tumor cells and inflammatory environment. In addition to using autophagy inhibitor and DNase I to control cancer recurrence, new strategies that inhibit NETosis may also be key to preventing metastasis. How autophagy changes the gene expression network of NETosis and the role of autophagy during cancer recurrence and metastasis are important questions that need to be answered in the future. Our results highlight the importance of pneumonia‐NETs in cancer metastasis and present potential therapeutic strategy for controlling cancer recurrence and metastasis.

## 5 MATERIALS AND METHODS

### 5.1 Ethics statement

Female BALB/c mice (6‐8 weeks old, 16‐18 g) were purchased from Beijing Vital River Laboratory Animal Technology Co, Ltd, maintained under specific pathogen‐free conditions and raised in the Central Animal Care Services of the Chinese Academy of Medical Sciences (CAMS) and Peking Union Medical College (PUMC). The animal experiments were approved by the Ethics Committee of Animal Care and Welfare of the Institute of Medical Biology, CAMS, and PUMC (Permit Number: SYXK (dian) 2010‐0007). All efforts were made to minimize animal suffering.

### 5.2 Bacterial culture and infection of mice

The clinical MDR *S. aureus* strain was isolated from the sputum smear of a patient at the First Affiliated Hospital of Kunming Medical University (Kunming, China). The bacterium was identified with a Vitek 32 system (BioMérieux, France) and further verified by 16S rDNA sequencing with the universal primers 27f and 1292R. *Staphylococcus aureus* was grown overnight with shaking in Luria‐Bertani (LB) medium at 37°C. The next day, the culture was subcultured in fresh LB medium for an additional 3‐4 h until the bacteria reached an OD600 value of 0.5. The supernatant was removed, and bacteria were collected by centrifugation at 5000 × *g* for 10 min. After washing twice with fresh sterile phosphate‐buffered saline (PBS), *S. aureus* was resuspended in PBS. Eight female mice were infected intranasally with 50 µL of a PBS solution containing 8 × 10^8^, 1.6 × 10^8^, and 3.2 × 10^7^ CFUs of live bacteria per mouse. The survival rate of unsacrificed mice was continuously monitored for 7 days and used as the endpoint.

### 5.3 Murine lung metastasis model and survival

To generate a murine lung metastasis model, the mouse breast cancer cell line 4T1 and melanoma cell line B16‐F10 were cultured in RPMI 1640/DMEM/F‐12 medium supplemented with 10% fetal bovine serum (FBS), 1% penicillin‐streptomycin, and 1% nonessential amino acids. Before injection, 4T1 and B16‐F10 cells were collected and washed twice with fresh sterile PBS. Mice were intravenously injected with a 200 µL 4T1 or B16‐F10 cell suspension containing a total of 1 × 10^5^ cells, and the mouse survival rate was monitored daily until all mice died.

### 5.4 Symptoms of neutropenia

To analyze the effect of neutrophils on metastasis, mice were intranasally injected twice with 50 µL cyclophosphamide at a final concentration of 1 mg/kg. Mouse lung BALF neutrophils were monitored by flow cytometry. Briefly, all cells in the BALF were collected and washed with PBS, cells were analyzed with Swiss‐Giemsa staining after cytospins, and flow cytometry was performed as previously reported.^28^

### 5.5 Preparation of tissue samples for histopathological analysis

Lung tissues were obtained, frozen in liquid nitrogen, embedded in optimal cutting temperature compound, and sectioned (5 µm thickness). Sections were stained with H&E and examined. To count the number of lung metastatic nodules, whole lung tissues were stained with Bouin's buffer, and the left lobe of the lung was sectioned and stained with H&E.

### 5.6 Flow cytometry

For the flow cytometry analysis of neutrophils, macrophages, NK cells, and T cells, cells were collected from lung BALF and washed with staining buffer. The flow cytometry protocol was performed as we previously reported.^42^ Briefly, PE‐labeled antimouse Gr‐1 and APC‐labeled antimouse CD11b were used as markers for neutrophils; FITC‐labeled antimouse CD3 and APC‐labeled antimouse NK1.1 were used as markers for NK cells; and FITC‐labeled antimouse CD45, APC‐labeled antimouse CD8, and PE‐labeled antimouse CD4 were used as markers for CD8/CD4 cells. All flow cytometry antibodies were purchased from BioLegend (USA). Cells were washed twice with staining buffer and stained with premixed antibodies at 4°C in the dark for 60 min. After staining, cells were washed twice with staining buffer and then resuspended in 100 µL staining buffer. Cells were analyzed by flow cytometry (BD Biosciences, Accuri C6), and data were analyzed using FlowJo software (FlowJo, LLC).

### 5.7 Real‐time quantitative polymerase chain reaction

Total RNA was isolated from cells and tissues using TRIzol (RNAiso, Takara) and purified according to the chloroform‐phenol extraction method. cDNA was reverse transcribed with a SureScript First‐stand cDNA Synthesis Kit (GeneCopoeia) and then detected with an All‐in‐One miRNA qRT‐PCR Detection Kit (GeneCopoeia). Real‐time quantitative polymerase chain reaction was performed on a Bio‐Rad CFX‐96 Touch Real‐Time Detection system. Primer sequences are listed in Table S1.

### 5.8 Visualization and quantification of NETs in vitro and in vivo

Neutrophils were separated from mouse PBMCs with neutrophil separation solution (TBD, China) and cultured in RPMI 1640 medium containing 10% FBS, 1% L‐glutamine, 1% nonessential amino acids, 100 U/mL penicillin, and 100 µg/mL streptomycin. Neutrophil purity was determined with cytospins and Swiss‐Giemsa staining. For the analysis of *S. aureus* infection‐induced NETs, the next day, neutrophils were collected, washed twice with PBS, and seeded onto polylysine‐coated slides (1 × 10^5^ cells per well) with cytospins. Two hundred microliters of fresh RPMI 1640 medium without antibiotics containing 5 × 10^6^ CFUs of *S. aureus* were added to the neutrophils and incubated for 6 h at 37°C in an atmosphere of 5% CO_2_. After stimulation, cells were washed twice and fixed with 4% paraformaldehyde for 15 min. Cells were blocked with 10% goat serum at room temperature for 30 min, and stained with anti‐MPO (ab208670, 0.489 mg/mL, 1:500; Abcam, UK) or anti‐H3cit (Abcam, ab5103,1:250) overnight at 4°C. Then, FITC‐labeled goat antirabbit IgG H&L (Abcam, ab6717, 1: 1000) was used; for lung tissues staining, Alexa Fluor 594‐labeled anti‐Ly6g (Biolegend, 127636, 1:1000) was used. Cells were washed and incubated with 4′,6‐diamidino‐2‐phenylindole (DAPI) for 15 min at room temperature. The cells were observed and imaged under a fluorescence microscope (Nikon, Japan). The NETs area was determined with ImageJ software.

### 5.9 Immunoblotting assay

To analyzed NETs formation in lung tissues, we harvested lung tissues after bacterial infection. Lung tissues were lysis with radio‐immunoprecipitation assay (RIPA) lysis buffer and separated with 12% sodium dodecyl sulphate‐polyacrylamide gel electrophoresis (SDS‐PAGE). The samples were transferred onto polyvinylidene fluoride or polyvinylidene difluoride (PVDF) membrane, which is blocked with 5% BSA. Then, the membrane is coated with anti‐H3cit (Abcam, ab5103,1:3000), anti‐HMGB1, and anti‐β‐actin overnight at 4°C. After washing, the membrane is coated with goat antimouse or antirabbit‐HRP for 1 h at room temperature. Protein bands were imaged with an enhanced chemiluminescence (ECL, Thermo Fisher Scientific, USA) according to the manufacturer's instructions. Image J was used to quantify the grayscale value of the immunoblot bands.

### 5.10 Immunofluorescence staining and microscopy

For MPO immunostaining, lung tissues were fixed in 4% paraformaldehyde for 15 min at room temperature. Lung tissues were washed three times with PBS and permeabilized with 0.1% Triton X‐100 at room temperature for 10 min. Then, lung tissues were blocked with 10% goat serum at room temperature for 30 min and stained with anti‐MPO (ab208670, 0.489 mg/mL, 1:500; Abcam, UK), anti‐H3cit (Abcam, ab5103,1:250), and 594‐labeled‐Ly6G overnight at 4°C. Lung tissues were washed and stained with a FITC‐labeled goat antirabbit secondary antibody (2 mg/mL, 1:1000, Abcam, UK) for 60 min at 37°C. Lung tissues were washed and incubated with DAPI for 15 min at room temperature. Lung tissues were observed and imaged under a fluorescence microscope (Nikon, Japan).

### 5.11 Cell viability assay

For the cell viability assay, neutrophils, which were cultured overnight in RPMI 1640 medium, were collected and resuspended in antibiotic‐ and FBS‐free DMEM without phenol red. Neutrophils were seeded into 96‐well plates at a density of 1 × 10^4^ cells per well at 100 µL/well. Then, the cells were infected with *S. aureus* at a gradient dose of 0, 5, 50, or 100 MOI. Cell viability was determined at 4, 6, 10, and 18 h according to the manufacturer's instructions. Briefly, sodium 3′‐[1‐(phenylaminocarbonyl)‐3,4‐tetrazolium]‐bis‐4‐methoxy‐6‐nitro) benzene sulfonic acid hydrate was dissolved in fresh DMEM and mixed with the electron‐coupling reagent PMS (N‐methyl dibenzopyrazine methyl sulfate) at a volume ratio of 50:1. After bacterial stimulation, neutrophils were treated with 50 µL XTT working reagent for 6 h. The formula mock cell absorbance [A450 nm – A620 nm] – test cell absorbance [A450 nm – A620 nm] was used to quantify dead cells.

### 5.12 Bacterial killing assay

To evaluate the effect of NETs on bacterial killing, neutrophils (1 million cells per well) in fresh medium were seeded into 24‐well plates and treated with the autophagy inhibitor 3‐MA (100 µM, Solarbio, China) and the agonist rapamycin (100 nM, MedChemExpress, Monmouth Junction, NJ, USA) for 6 h before 10 MOI of *S. aureus* infection. After 6 h of stimulation, cell supernatants were collected and plated onto LB agar plates in a serial dilution and incubated overnight to determine the number of CFUs.

### 5.13 LDH release assay

Cell culture supernatants were collected under different processing conditions, and LDH activity was measured with the LDH cytotoxicity assay kit according to the manufacturer's instructions. Briefly, after stimulation, cells were treated with LDH release reagent for 1 h, and 120 µL cell supernatant was moved to a new 96‐well plate. Twenty microliters of lactic acid solution, INT solution, and enzyme solution were mixed to make the working solution. Cells were treated with 60 µL working solution and slowly shaken at room temperature for 30 min in the dark. The formula A_490 nm_ – A_620 nm_ was used to quantify LDH release.

### 5.14 Statistical analysis

GraphPad Prism 8.0 (GraphPad Software, Inc., La Jolla, CA, USA) was used for statistical analyses. All data are given as the mean ± standard error of the mean (SEM). Statistical analyses between two groups were performed using a two‐tailed Student's *t*‐test, one‐way analysis of variance was used for multiple comparisons, and a nonparametric long‐rank test was used to compare survival rates. *P*‐values ≤ .05 were considered significant; ^*^ indicates a *P*‐value < .05 (considered significant), ^**^ indicates a *P*‐value < .01, ^***^ indicates a *P*‐value < 0.001, and ^****^ indicates a *P*‐value < .0001.

## CONFLICT OF INTEREST

The authors declare no conflict of interest.

## Supporting information

Supplemental Figure 1. Depleting neutrophils suppress breast cancer lung metastasis.
(A) A schematic diagram of neutrophil depleting suppressed cancer metastasis. After cancer injection, 5 µg/mice anti‐Ly6G were injected for 5 time every 2 days.
(B) Representative images of lung tissues fixed with Bouin's buffer.
(C) The results of lung metastatic nudes.

Supplemental Figure 2. NETs formation was represented as a tumor development processes‐dependent manner.
(A) Indirect fluorescence assay (IFA) of NET formation in tumor tissues with different volume. Small tumor diameter < 5 mm; Medium diameter < 15 mm; Large diameter > 20 mm.
(B) Indirect fluorescence assay (IFA) of NET formation with the staining with MPO *in vitro*.
(C) The quantification analysis of NET areas per section.
The data represent three independent experiments and are shown as the mean ± SEM. ^**^p < 0.01, ^***^p < 0.001, ^****^p < 0.0001.

Supplemental Figure 3. NETs formation was represented in lung metastatic nudes.
(A) Indirect fluorescence assay (IFA) of NET formation in lung metastatic nudes.
(B) The quantification analysis of NET areas per section.
The data represent three independent experiments and are shown as the mean ± SEM. ^*^p < 0.05.

Supplemental Figure 4. Neutrophils were recruits in lung tissues after bacterial infection.
(A) Representative images of FCM assay. Ly6G‐PE and CD11b‐APC were used for BALF neutrophils identification.
(B)The quantification analysis of neutrophils in BALF.
The data represent two independent experiments and are shown as the mean ± SEM. ^****^p < 0.0001.

Supporting Information.
