# Supplementary figures and images for: Pulmonary Staphylococcus aureus infection regulates breast cancer cell metastasis via neutrophil extracellular traps (NETs) formation
Source: MedComm (2020). 2020 Jul 30;1(2):188–201. doi: 10.1002/mco2.22 (PMC8491238; doi:10.1002/mco2.22)

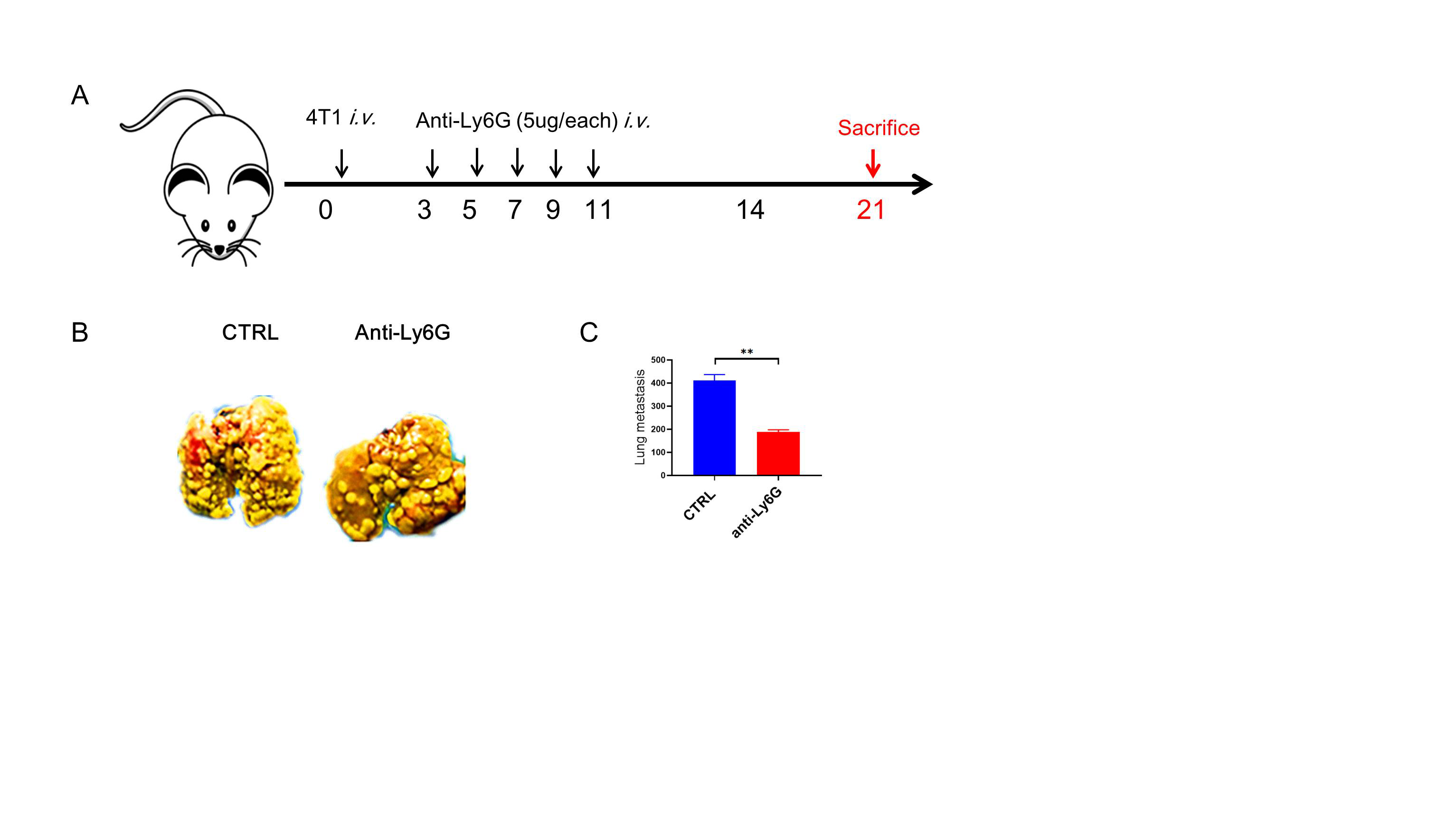

Supplement: Supplementary file 1 — Supplemental Figure 1. Depleting neutrophils suppress breast cancer lung metastasis. (A) A schematic diagram of neutrophil depleting suppressed cancer metastasis. After cancer injection, 5 µg/mice anti‐Ly6G were injected for 5 time every 2 days. (B) Representative images of lung tissues fixed with Bouin's buffer. (C) The results of lung metastatic nudes. [file MCO2-1-188-s002.tif]

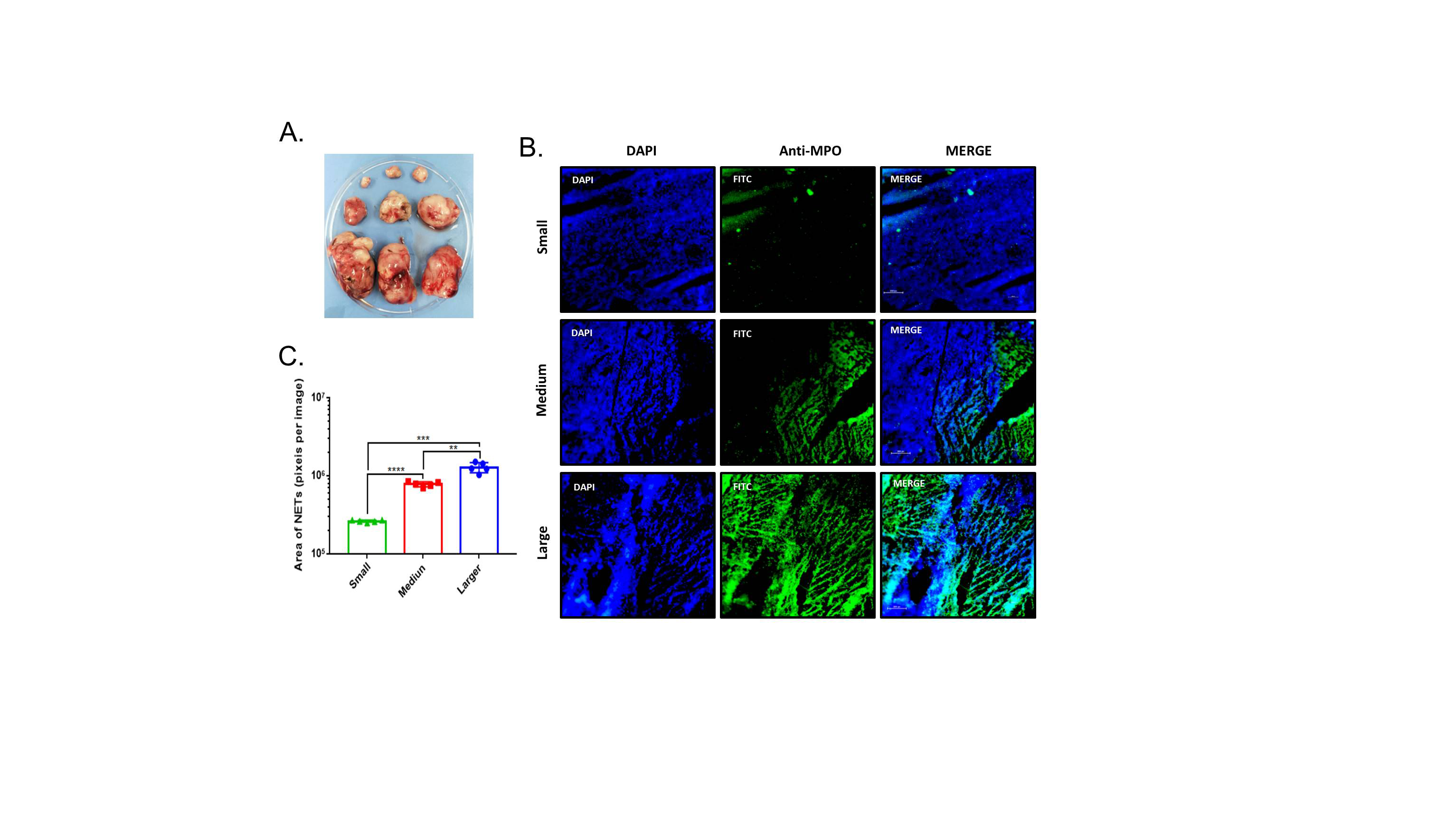

Supplement: Supplementary file 2 — Supplemental Figure 2. NETs formation was represented as a tumor development processes‐dependent manner. (A) Indirect fluorescence assay (IFA) of NET formation in tumor tissues with different volume. Small tumor diameter < 5 mm; Medium diameter < 15 mm; Large diameter > 20 mm. (B) Indirect fluorescence assay (IFA) of NET formation with the staining with MPO in vitro. (C) The quantification analysis of NET areas per section. The data represent three independent experiments and are shown as the mean ± SEM. **p < 0.01, ***p < 0.001, ****p < 0.0001. [file MCO2-1-188-s005.tif]

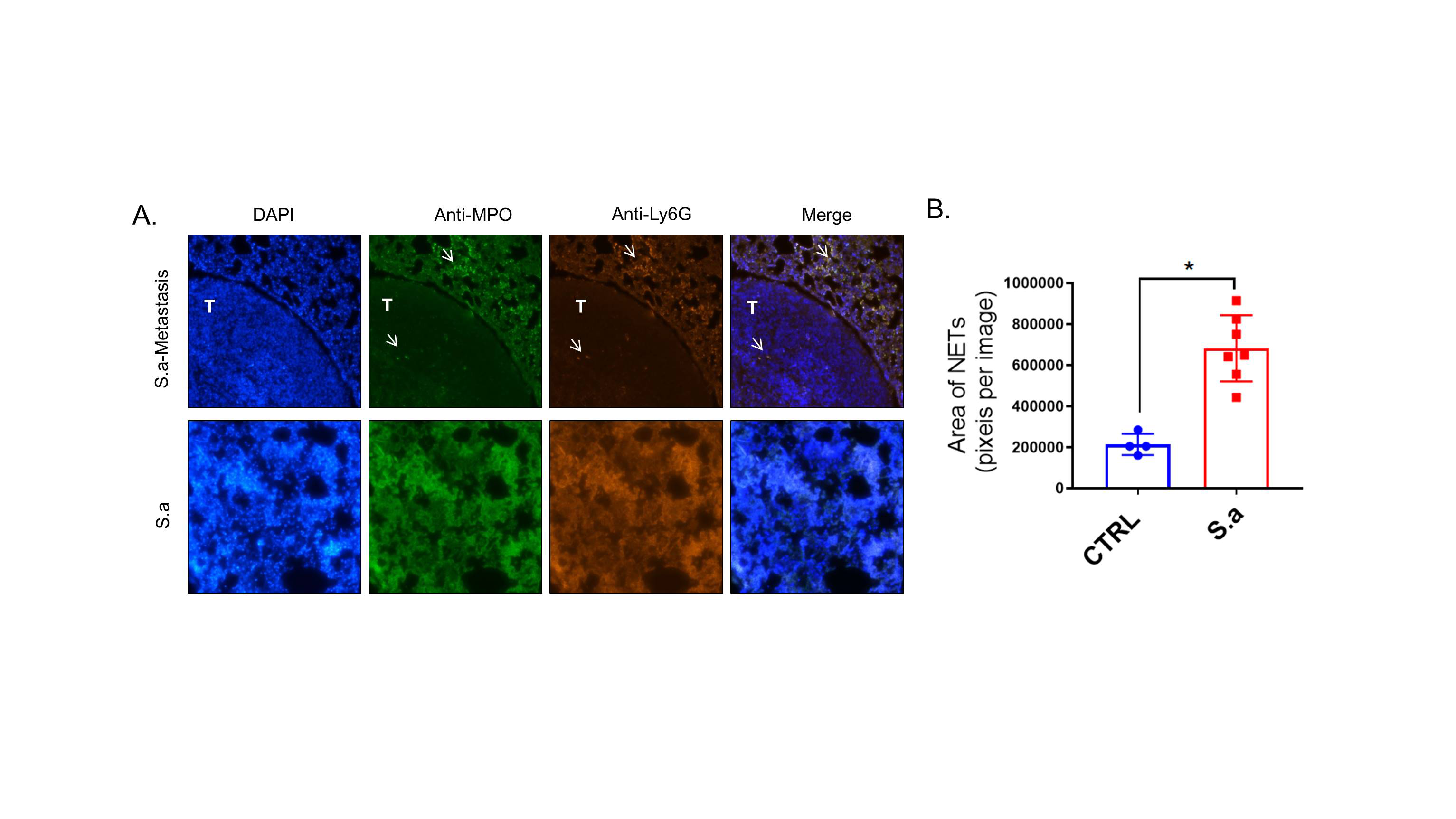

Supplement: Supplementary file 3 — Supplemental Figure 3. NETs formation was represented in lung metastatic nudes. (A) Indirect fluorescence assay (IFA) of NET formation in lung metastatic nudes. (B) The quantification analysis of NET areas per section. The data represent three independent experiments and are shown as the mean ± SEM. *p < 0.05. [file MCO2-1-188-s001.tif]

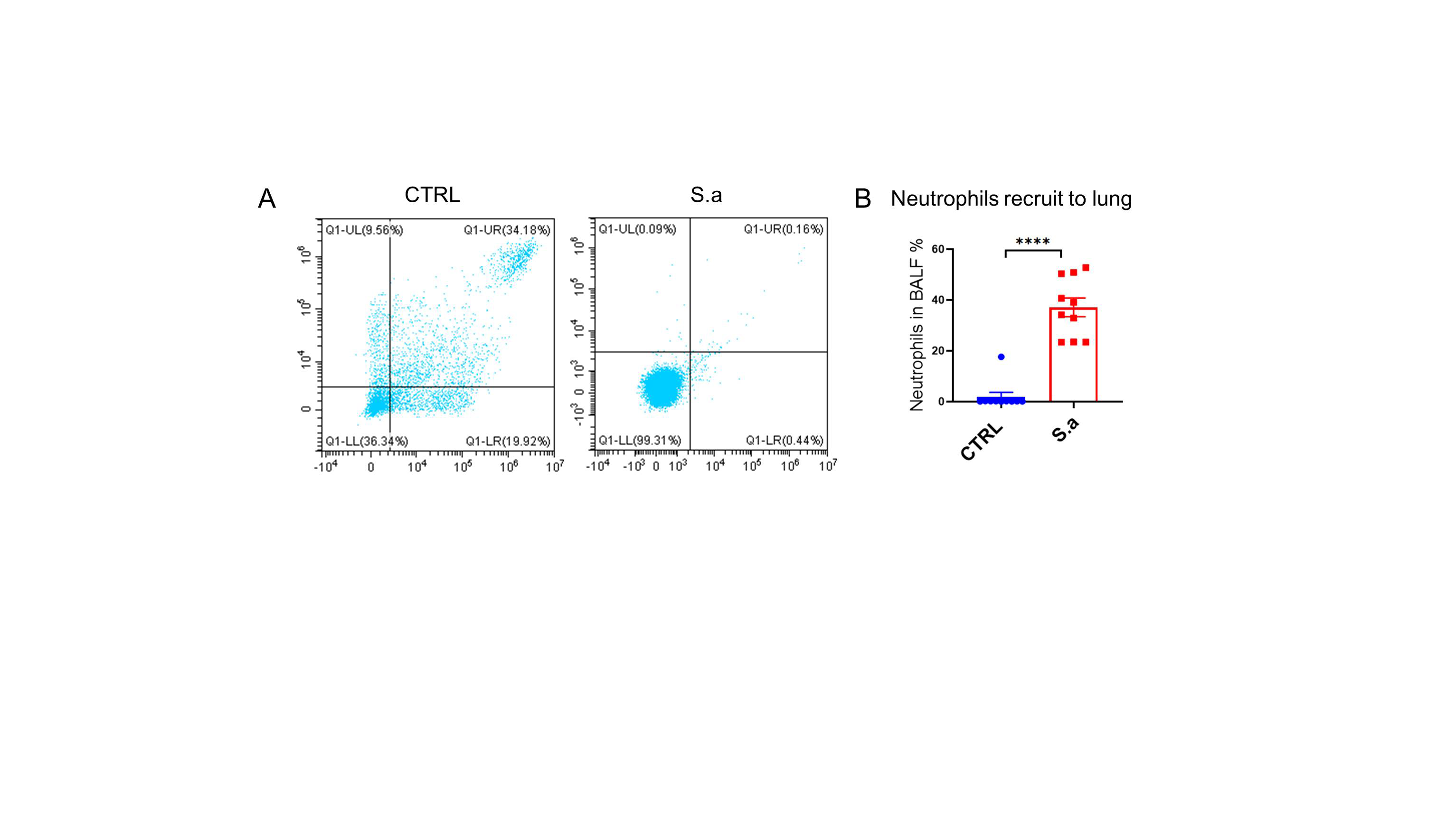

Supplement: Supplementary file 4 — Supplemental Figure 4. Neutrophils were recruits in lung tissues after bacterial infection. (A) Representative images of FCM assay. Ly6G‐PE and CD11b‐APC were used for BALF neutrophils identification. (B)The quantification analysis of neutrophils in BALF. The data represent two independent experiments and are shown as the mean ± SEM. ****p < 0.0001. [file MCO2-1-188-s003.tif]
